# Religiousness and preoperative anxiety: a correlational study

**DOI:** 10.1186/1744-859X-6-17

**Published:** 2007-06-29

**Authors:** Masoomeh Aghamohammadi Kalkhoran, Mansoureh Karimollahi

**Affiliations:** 1 Faculty member of Ardabil Medical Sciences University, Ardabil, Iran

## Abstract

**Background:**

Major life changes are among factors that cause anxiety, and one of these changes is surgery. Emotional reactions to surgery have specific effects on the intensity and velocity as well as the process of physical disease. In addition, they can cause delay in patients recovery. This study is aimed at determining the relationship between religious beliefs and preoperative anxiety.

**Methods:**

This survey is a correlational study to assess the relationship between religious beliefs and preoperative anxiety of patients undergoing abdominal, orthopaedic, and gynaecologic surgery in educational hospitals. We used the convenience sampling method. The data collection instruments included a questionnaire containing the Spielberger State-Trait Anxiety Inventory (STAI), and another questionnaire formulated by the researcher with queries on religious beliefs and demographic characteristics as well as disease-related information. Analysis of the data was carried out with SPSS software using descriptive and inferential statistics. Results were arranged in three tables.

**Results:**

The findings showed that almost all the subjects had high level of religiosity and moderate level of anxiety. In addition, there was an inverse relationship between religiosity and intensity of anxiety, though this was not statistically significant.

**Conclusion:**

The results of this study can be used as evidence for presenting religious counselling and spiritual interventions for individuals undergoing stress. Finally, based on the results of this study, the researcher suggested some recommendations for applying results and conducting further research.

## Background

It is widely accepted that people awaiting surgery experience anxiety [1]. Anticipation of postoperative pain, separation from the family, loss of independence as well as fear of surgery and death are factors triggering symptoms of preoperative anxiety.

Incidences of preoperative anxiety have been reported in 11% to 80% of adult patients. Consequently, there has been a growing interest in the possible influences of preoperative anxiety on the course and outcomes of surgical treatments, as well as in the study of anxiety-reducing interventions [2].

Most surgeons postpone operations in cases with high anxiety [3]. Therefore, the importance of anxiety in surgery patients shows the necessity of its prevention.

When physical illness strikes, religion and spirituality become important factors in coping. This may be particularly true for hospitalized patients, who must cope not only with unpleasant physical symptoms but also with the stress of hospitalization. Hospitalization can trigger underlying conflicts regarding separation and loss and threaten one's sense of control and adequacy, because patients undergoing surgery must abandon their usual roles in society, take on a more dependent role, and confront the unknown. Likewise, confinement to a hospital bed and hospital routines restrict mobility, limit stimulation, and often assault the patient's sense of competence. Religious or spiritual beliefs may help patients to cope with these stressful experiences [4].

Anxiety is the second most commonly studied disorder with respect to the relationship of mental health and religion. A recent article of Shreve-Neiger and Edelstein cited by Flannelly et al. reviewed 17 studies on religion and general anxiety published since 1962. Most of the studies found a negative relationship between religion and anxiety level, five found a positive relationship, and four found no relationship at all. The strongest evidence for a negative relationship between anxiety and religiosity came from studies of community-dwelling individuals, but the results were far from conclusive [5]. In addition, Mohr reported more than 850 studies that examined the relationship between religious involvement and various aspects of mental health [6]. These aspects consist of depressive symptoms and suicide. For example, twenty-four studies have found that religiously involved people had fewer depressive symptoms and less depression [7]. In addition, Gartner, Larson and Vacher-Mayberry (1990) conducted a review of empirical studies on the relationship between religious commitment and mental health, and found that religious commitment was related inversely to suicide in 13 of 16 (81%) reviewed studies [8].

However, cross-sectional studies have yielded both significant [9] and insignificant [10-12] associations between different indicators of religiosity and a lower prevalence of depression in various populations.

Therefore, considering the controversial findings, we decided to do a systematic study to determine the relationship between preoperative anxiety and religious beliefs in the context of an Iranian community that puts great emphasis on religious beliefs and using the author's experience in dealing with anxious patients who use religious practices to cope with their anxiety.

## Methods

This is a correlational study aimed to determine the relationship between religiosity and preoperative anxiety in patients hospitalized in surgical units of educational hospitals in Ardabil, Iran. The ethical approval for doing this research was given by the research committee of Ardabil Medical Sciences University.

The study population were all patients who were candidates for abdominal, orthopaedic, and gynaecologic surgery, and the subjects consisted of 150 patients chosen using the convenient method of sampling. Before research was initiated, the participants read and signed the consent form that offered the aims of study and confirmation about their anonymity, their right to reject taking part in the study any time they wished, and the assurance that their treatment and care would not be affected by this research. The sample volume was calculated using the following formula:

N = z^2^c.s^2^/e^2^

z = 1.96 α = 0.05 e = 1.8

Variance was calculated (115.45) using pilot study.

Inclusion criteria for subjects of this study were:

1. Must be above 15 years of age

2. Must be undergoing surgery for the first time

3. Must have had no endocrine and metabolic disorders and be in a stable physical condition as determined by their medical records

4. Must have no known mental disorders

5. Must have literacy to respond to questions

6. Must have no pain during time of data collection

7. Must have volunteered to participate in the study

Exclusion criteria were:

1. Have fever at the time of data gathering

2. Have special surgeries like heart and neurological ones

For collecting data, the Spielberger scale (STAI) was used to determine anxiety intensity because of its high validity and reliability and its ability to determine state and trait anxiety, which were important for this study. The inventory comprised 40 questions translated into Farsi. The reliability (r = 0.97) and validity of this translated version has been cited in Persian journals. The anxiety scale ranged from 40 points for no anxiety to a maximum of 160 points [13].

In addition, a questionnaire formulated by the researcher was used to assess religious beliefs. This comprised questions regarding belief in God and in life after death, the effect of religious beliefs and behaviours in life, freedom, patience, hope, and importance of religion in life. These items were included following extensive search of Islamic literature and considering the views of Islamic scholars. This tool was validated by content validity method using the views of expert colleagues. After reviewing their comments, appropriate changes were made. Some of the reviewers agreed with the inclusion of religious practices in the questionnaire, but these were not included because of their irrelevance to the topic.

For determining reliability of tools, the split-half method and Spearman-Brown predictive test were conducted on pilot study results (r = 0.94).

It should be mentioned that the anxiety inventory questionnaire has been used in many studies [14-16] and the mean of its reliability has been reported at 97%, so it seems there was no need to determine its reliability again in this study.

For analysis, questionnaires were coded and entered into SPSS software and analyzed using descriptive and inferential (χ^2 ^and exact fisher tests) statistics.

## Results

Findings showed that the majority (68%) of patients were in the age range of 15–20 years (mean = 26.54 ± 9.26). About 73.3% were female and 71.3% of participants were married. The majority had no children (45.3%), 38% had high school education, and 62.7% were urban residents. For a majority of patients (69.3%), hospitalization period was less than 24 hours. Approximately 60% were being hospitalized for abdominal surgery.

In addition, almost 66.7% had a high religiosity score (mean = 115.107 ± 11.77), as shown in Table 1.

**Table 1:** Level of Religiosity

Level of Religiosity	Number	Percent
Low	***0***	***0***
Moderate	***50***	***33.3***
High	***100***	***66.7***
Total	***150***	***100***
Mean	**84.58**	
SD	**16.95**	

The results also showed that the majority (66.7%) of patients had moderate anxiety (mean = 84.58 ± 16.95), as shown in Table 2.

**Table 2:** Intensity of Anxiety

Level of Anxiety	Number	Percent
Mild	***58***	***38.7***
Moderate	***82***	***54.7***
Severe	***10***	***6.7***
Total	***150***	***100***
Mean	***115.107***	
SD	***11.77***	

Regarding correlation of religiosity and anxiety, the results did not show a significant relationship, but there was a reverse correlation between them (r = -0.05). What is more significant is the finding that patients with low religiosity had moderate to severe anxiety (Table 3).

**Table 3:** Relationship between Religiosity and Anxiety

Result	Total		Moderate and severe		Mild		*Anxiety*
	
	***p***	***n***	***P***	***n***	***p***	***n***	***Religiosity***
χ^2 ^= 0.48	***100***	***9***	***77.78***	***7***	***22.22***	***2***	***Low and moderate***
P = 0.49 R=-0.05	***100***	***141***	***60.28***	***85***	***39.71***	***56***	***High***
Not significant	***100***	***150***	***61.33***	***92***	***38.67***	***58***	***Total***

In determining the relationship between sex and level of religiosity of patients, findings showed that females had a higher level of religiosity. This positive relationship was also applicable to marriage state and religiosity, in that married patients had a higher level of religiosity.

## Discussion

Although the findings of this study showed a reverse relationship between religiosity and anxiety, the relationship was not significant statistically. Religious beliefs help patients make sense of their medical conditions and may enable them to better integrate health changes into their lives. Religious practices can help to relax, distract, and counteract the effects of loneliness and isolation that is so prevalent among patients.

In Koenig's study about religion, spirituality, and health in medically-ill and hospitalized older patients, religiousness and spirituality consistently predicted greater social support, fewer depressive symptoms, better cognitive function, and greater cooperativeness (P = 0.01 to P = 0.0001), while the relationship of physical health with these same factors was weaker, although similar in direction. Furthermore, organizational religious activity predicted better physical functioning and observer-rated health, as well as less-severe illnesses. Intrinsic religiosity also tended to be associated with better physical functioning, while observer-related religiousness and observer-related spirituality were associated with less-severe illnesses and lesser medical co-morbidity (all P = 0.05). What is more, patients categorizing themselves as neither spiritual nor religious tended to have worse self-rated and observer-rated health and greater medical co-morbidity [4].

Although the majority of studies support the existence of a relationship between religious beliefs and anxiety, other studies have shown a weak relationship between them [14-16].

The feeble relationship found in this study may have been due to several reasons. One reason may be that, other causes of anxiety such as biological, environmental, and intrinsic factors may have had stronger influence than religious beliefs (the one among many factors) on the patients' state of mind. This therefore supports the holistic view about human beings, that man consists of bio-psycho-socio-spiritual dimensions and no single dimension can predict human behaviour. Secondly, although surgery causes high anxiety in patients, our patients have had moderate to low levels of anxiety. This incongruence may be due to the effect of high level of religiousness in our patients.

## Conclusion

This study showed a non-significant relationship between anxiety and religiosity. The results of this study can be used as evidence for presenting religious counselling and spiritual intervention for individuals under higher levels of stress. In addition, these results may be useful in various aspects of patient care, including education of medical sciences students, treatment and research. The present study could be pursued in the following directions:

1. This study was conducted on an Islamic population. Further studies can be conducted in other religious communities and among atheists and agnostics.

2. This study was carried out in the Iranian setting, so it is recommended that similar studies be conducted in other countries.

3. This study investigated the relationship between religiosity and preoperative anxiety. We recommend that further studies investigate the relationship between other anxiety disorders and religiosity.

4. This study was about religious beliefs and anxiety. The relationship between religious practices and anxiety level could be another worthwhile line of research.

5. Because hospitalization naturally causes anxiety in people, we recommend doing similar research among ordinary people in a community.

6. Because of the limited number of our sample (150), it is recommended that this research be conducted among a larger sample.

7. Because of the unavailability of standard questionnaires about religious beliefs in the Islamic context, it is recommended that further studies be carried out using a standard questionnaire, if available.

### Limitations

1. Because of limitation of time, we could not obtain a larger sample.

2. There was no standard questionnaire on religious beliefs of Muslim patients.
